# Engineering an All-Biobased Solvent- and Styrene-Free Curable Resin

**DOI:** 10.1021/acspolymersau.3c00015

**Published:** 2023-10-03

**Authors:** Samson Afewerki, Ulrica Edlund

**Affiliations:** Fibre and Polymer Technology, KTH Royal Institute of Technology, SE 100 44 Stockholm, Sweden

**Keywords:** curable resin, biobased, catalysis, renewable feed-stock, biomass, environmental challenges

## Abstract

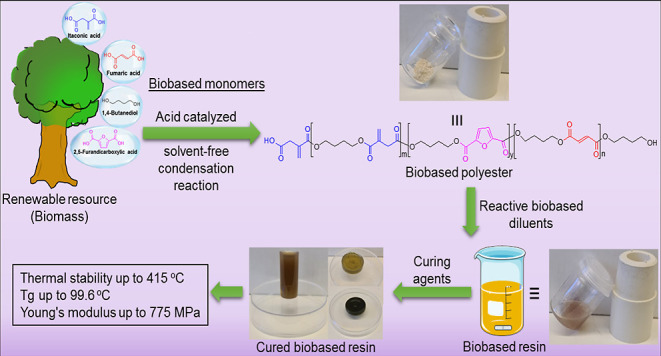

The sustainable production of polymers and materials derived from renewable feedstocks such as biomass is vital to addressing the current climate and environmental challenges. In particular, finding a replacement for current widely used curable resins containing undesired components with both health and environmental issues, such as bisphenol-A and styrene, is of great interest and vital for a sustainable society. In this work, we disclose the preparation and fabrication of an all-biobased curable resin. The devised resin consists of a polyester component based on fumaric acid, itaconic acid, 2,5-furandicarboxylic acid, 1,4-butanediol, and reactive diluents acting as both solvents and viscosity enhancers. Importantly, the complete process was performed solvent-free, thus promoting its industrial applications. The cured biobased resin demonstrates very good thermal properties (stable up to 415 °C), the ability to resist deformation based on the high Young’s modulus of ∼775 MPa, and chemical resistance based on the swelling index and gel content. We envision the disclosed biobased resin having tailorable properties suitable for industrial applications.

## 1 Introduction

The current environmental and greenhouse gas emissions challenges promote the advancement of renewable technologies and products.^1^ The employment of biobased feedstocks is instrumental in battling the environmental challenges we are facing.^2–8^ These renewable resources will have tremendous importance in their usage for fabricating biobased polymers and materials for various unmet needs and substitution in existing applications.^9,10^ In this context, resins such as vinyl esters used as high-performance thermoset resins are very useful components for various applications such as coatings,^11^ electrical insulation systems, buildings, and matrices in fiber-reinforced composites for a range of engineering applications.^12^ In 2020, the worldwide market size of vinyl ester was assessed at $1.1 billion, and it is anticipated to grow to $2.1 billion by the year 2030.^13^ These types of resins have several advantages, such as favorable mechanical and thermal properties,^14^ durability, low weight, and cost,^15^ ease of handling, and abundance.^16^ Nevertheless, they have several drawbacks that limit their future applications, such as being nonbiodegradable,^17^ derived from petroleum-based monomers,^18^ and hard to recycle.^19^ Furthermore, many curable resins commercially used today contain high concentrations of styrene (>40% wt %) as the reactive diluent.^20^ However styrene has demonstrated several environmental and health problems, e.g., odor issues, being a hazardous air pollutant, and volatility.^21^ Some reports suggest that styrene is a potential human carcinogenic.^22,23^ In fact, the global styrene market is enormous, with an estimation of $53.12 billion in 2022 and expected to grow to $97.31 billion by 2032.^24^ Therefore, the development of biobased, nonvolatile reactive diluents with styrene-like performance is gaining increasing interest.^25^ Bisphenol-A is another very common fossil-based monomer used to fabricate vinyl ester resins but is harmful to health and the environment (e.g., endocrine-disruptive).^26,27^ Therefore, developing more sustainable technologies and products to overcome the above-mentioned challenges is essential and of great interest. Here, plant biomass has the potential to be one of the most renewable feedstock.^28^ It consists of the vital components cellulose, hemicellulose, and lignin, which can provide very useful monomers for further processing.^29^ Various physical,^30^ chemical,^31,32^ and biological processes^33,34^ have been developed to extract useful biobased monomers from lignocellulosic biomass.^35–37^

The family of polyesters has several advantages in providing macromolecular components in thermoset resins, such as facile preparation,^38^ low cost, potential biodegradability,^39^ and tunable properties.^40^ There have been several reports demonstrating the development of entire biobased resins; for instance, Brännström et al. used itaconic acid (IA), succinic acid (SA), and 1,4-butanediol (BD) to prepare curable polyesters. The obtained biobased polyester demonstrated a low melting point (∼45 °C) and a very low glass transition temperature (*T*_g_ = −31 to −38 °C).^41^ Nevertheless, the cured resin demonstrated increased *T*_g_ ∼ 12 to ≤100 °C. Dai and coauthors prepared a biobased polyester from the monomers IA, 2,5-furandicarboxylic acid (2,5-FDCA), SA, and 1,3-propanediol (PD), and the final resin was obtained by adding the reactive diluent guaiacol methacrylate.^42^ The cured resin demonstrated thermal stability up to 330 °C, *T*_g_ of 73.5–141.7 °C, and a flexural strength of 41.9–116.8 MPa. Moreover, a biobased resin combining curable polyester made from IA and PD with reactive diluents such as dialkyl itaconates such as dimethyl itaconate has also been reported.^43^ The cured resin showed a *T*_g_ of 65–118 °C, a storage modulus of 0.37–1.4 GPa, and a stress-at-break between 21 and 54 MPa.

**Figure 1:**
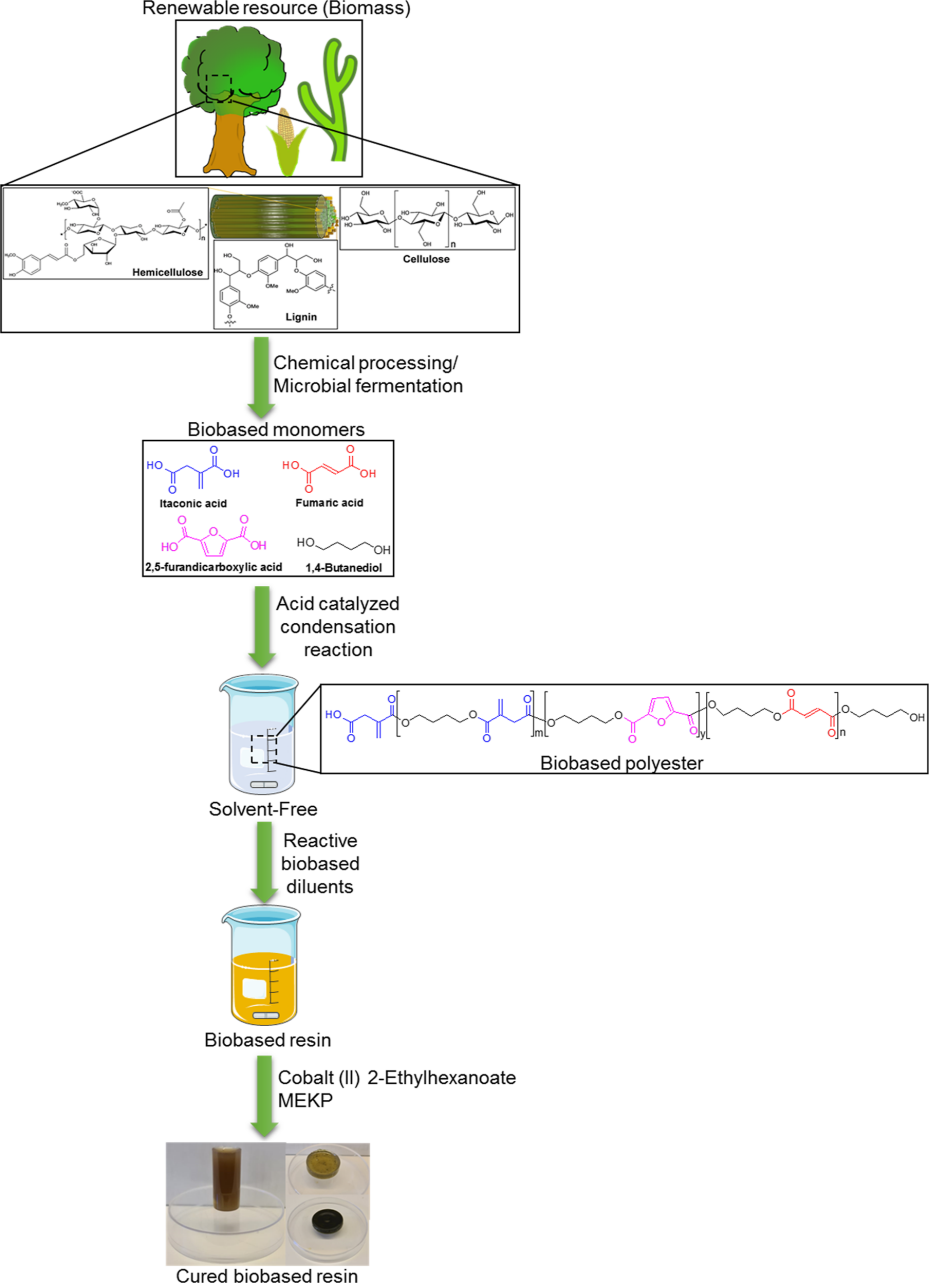
Schematic presents the overall chemical strategy explored in this work. The biobased monomers can be extracted from the renewable resource biomass containing the vital components cellulose, hemicellulose, and lignin through various processes such as chemical and microbial fermentation. Subsequent solvent-free acid-catalyzed condensation reaction provides the biobased polyester. Combining the polyester with biobased reactive diluents and further curing provides an all-biobased solvent- and styrene-free cured resin.

In our quest to develop environmentally friendly technologies and products^44,45^ and create alternative technological solutions to tackle vide supra challenges, we envisioned designing an all-biobased solvent- and styrene-free curable resin through a careful rational design. We hypothesize that a novel combination of IA, fumaric acid (FA), and 2,5-FDCA, with BD as the linker, will provide the curability, rigidity,^46^ and thermomechanical properties^47^ needed to design an all-biobased resin with similar or improved properties to a commercially available equivalent. To get a quick understanding of the differences in mechanical properties between our devised cured resin and commercially available resin, we performed initial tensile tests according to the ASTM D638 standard. The rational design, as depicted in Figure 1, starts with the preparation of a curable biobased polyester. All of the selected monomers can be extracted from renewable feedstocks such as biomass through various processes^48^ and have shown a significant increase in usage in the scientific community (Figure 1).^49,50^ The biobased polyester was synthesized through a green acid-catalyzed and solvent-free condensation reaction.^51^

The use of solvents in resins to dissolve the various components within the resin generally does not add any properties to the final resin and results in waste;^52^ therefore, having a neat mixture is desirable. Moreover, the optimal viscosity of the final resin is very important in the processing of resins.^53,54^ In this context, void defects impacted by the viscosity of the resin are one of the most common challenges affecting the mechanical performance and durability of the final product.^55^ Additionally, the resin viscosity impacts the outcome of mixing with reinforcement and the ability of the material to stay in the desired place during the handling.^56^ Therefore, the final all-biobased curable resin devised herein includes the unsaturated polyester in combination with a biobased reactive diluent and a reactive biobased viscosity enhancer (Figure 1).^25,57,58^ The use of these two important diluents will allow a solvent-free process and provide the final resin with a tunable viscosity for various industrial applications. The performance of the devised biobased resin was further compared to the widely used commercially available epoxy bisphenol A vinyl ester urethane (VER) containing styrene (<50 wt %).^59^

## 2 Results and Discussion

**Scheme 1:**
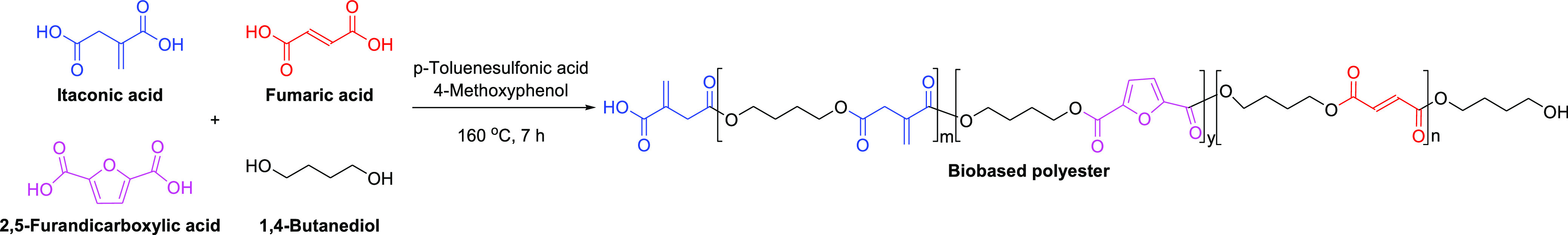
Synthesis of the Biobased Polyester Through an Acid-Catalyzed Condensation Reaction

**Figure 2:**
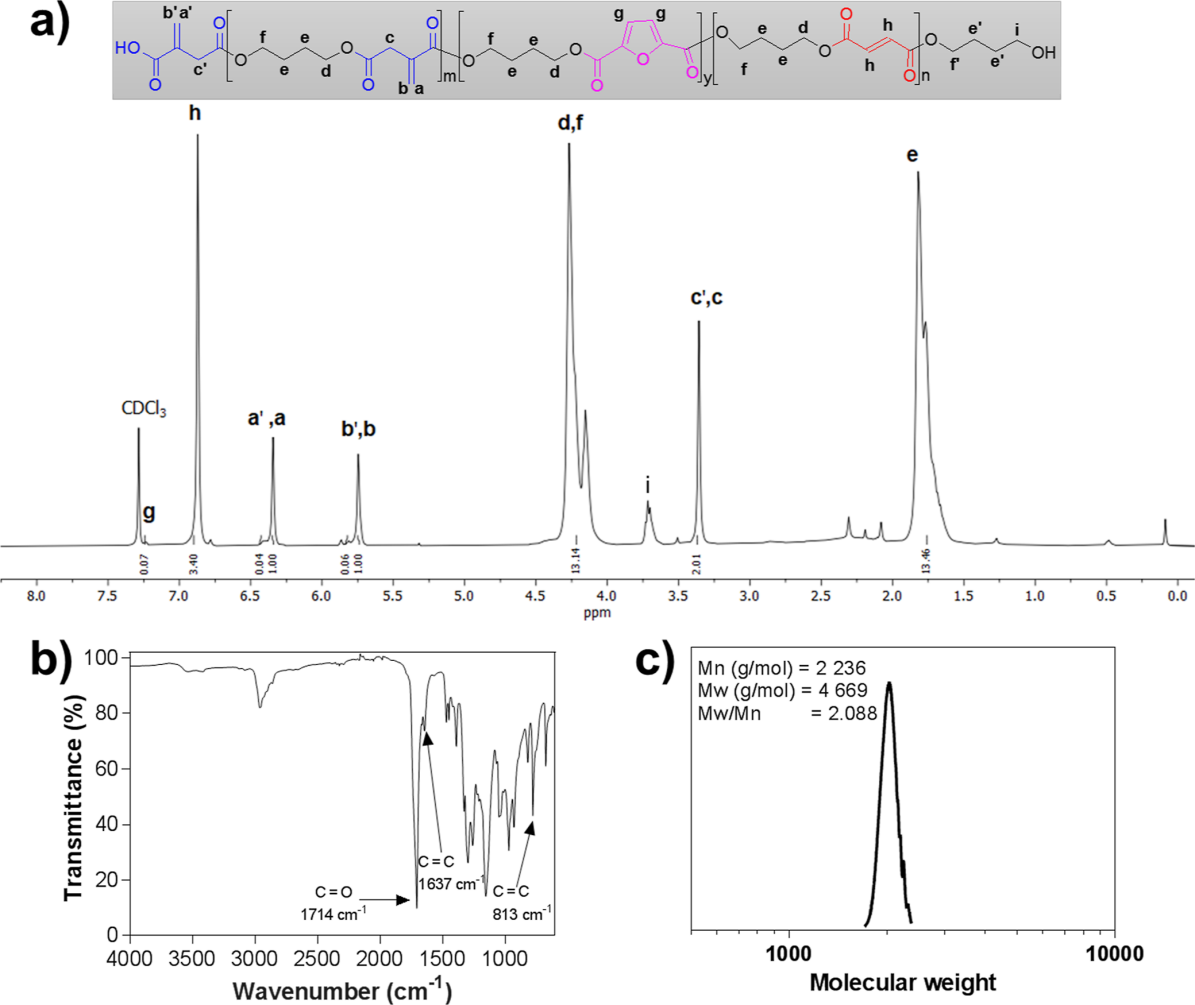
Characterization of the prepared biobased polyester. (a) Proton nuclear magnetic resonance (^1^H NMR) spectrum, (b) FTIR spectrum, and (c) SEC chromatogram.

The biobased polyester was prepared through an acid-catalyzed condensation reaction using the monomers IA, FA, 2,5-FDCA, and BD in the presence of the acid catalyst *p*-toluenesulfonic acid and mixed at 160 °C for 7 h (Scheme 1). The success of the reaction was confirmed through proton nuclear magnetic resonance spectroscopy (^1^H NMR) analysis, where the various components could be identified (Figure 2a). However, some minor traces from the monomers, such as 1,4-butanediol, could also be observed in the spectrum, for instance at 3.5, 2.2, and 2.1 ppm, as have been detected in previous reports (Figures S1–S4).^35^ Moreover, the NMR spectrum also showed small traces of additional peaks at 6.75 and 2.28 ppm that correspond to the mesaconic moiety (less than 4%, calculated by the ratio of the olefinic protons of itaconic and mesaconic acid from the ^1^H NMR spectrum), which is the isomerization product of itaconic acid formed at high temperatures.^60^ The Fourier transform infrared spectrometry (FTIR) analysis further corroborated the successful synthesis and the peaks corresponding to the carbonyl group (C=O) at 1714 cm^–1^ and a double bond (C=C) at 1637 and 813 cm^–1^ (Figure 2b). The weight-average molecular weight (*M*_w_) of the polyester was ∼4700 g/mol with a dispersity (*D̵* = *M*_w_/*M*_n_) of 2.09, as determined through size exclusion chromatography (SEC) (Figure 2c).

**Figure 3:**
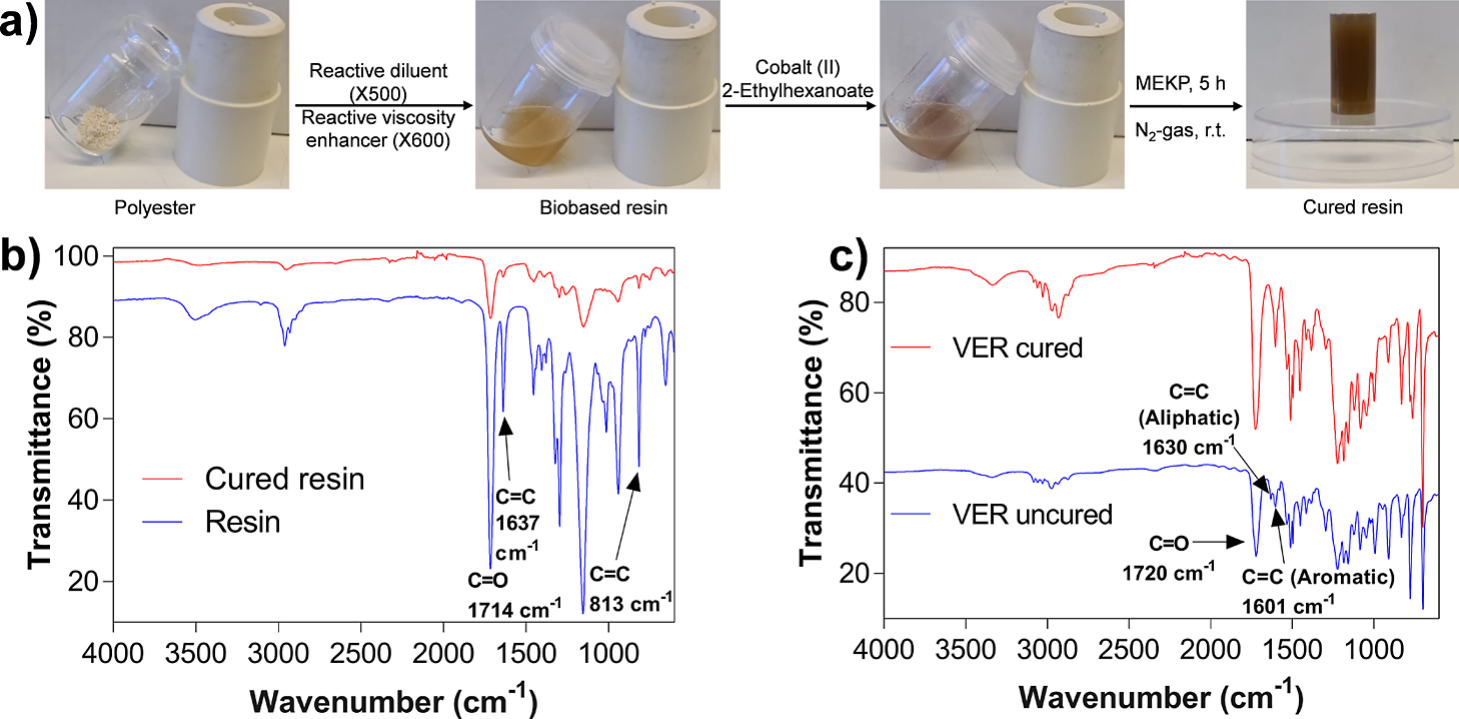
(a) Scheme demonstrating the preparation and curing of the biobased resin. (b) FTIR spectrum spectra of the resin and cured resin used to calculate the degree of cross-linking (DC). (c) FTIR spectra of the epoxy bisphenol A vinyl ester urethane (VER) and cured VER used to calculate the DC.

The fabrication and curing of the biobased resin are presented in Figure 3a. Initially, the polyester was mixed with reactive diluent X500 and reactive viscosity enhancer X600. Subsequently, the accelerator cobalt(II) 2-ethylhexanoate was added to the biobased homogeneous resin mixture. Afterward, the catalyst and radical initiator methyl ethyl ketone peroxide (MEKP) was added to initiate the curing, which, after 5 h of incubation, provided the cured biobased resin (Figure 3a). A systematic screening evaluation was performed on the curing by altering the concentrations of the cobalt accelerator and MEKP catalyst and the amount of polyester (Table 1). Using 0.5 g/mL of the polyester made the mixture too viscous, and it was difficult to obtain a homogeneous mixture (Table 1, entry 1). Nevertheless, using a 0.25 g/mL concentration provided a homogeneous solution. Increasing the concentration of the MEKP compared to the cobalt accelerator resulted in a higher curing efficiency (Table 1, entries 3, 4, and 6). These results are in accordance with previous reports, where having the right balance between the cobalt accelerator and the MEKP catalyst is crucial to obtaining successful curing.^61^ For instance, having a too high concentration of the accelerator can lead to deactivation of the reactive MEKP. In our case, using 0.25 wt % of the accelerator and 5.0 wt % of the MEKP generated the highest degree of cross-linking (DC) of 65% (Table 1, entry 7), which was determined through FTIR analysis (Figure 3b). Next, we evaluated the curing of the commercial VER using the two parameter settings that gave the best results of the ones studied (Table 1, entries 6 and 7). The curing almost went to full completion for the VER resin, providing a DC of 97–98% (Figure 3c, Table 1 entries 8 and 9). The analysis of swelling index (SI) and gel content (GC) was performed to gain a deeper and further understanding of the degree of curing and quality of the cured resin. The biobased cured resin demonstrated SI of 0.07% and GC of 98.7%, while the cured VER resin showed an SI of 35.5% and GC of 94.0%. The lower SI and higher GC of the biobased resin advocate for a higher degree of cross-linking, better mechanical integrity, higher thermal stability, and better chemical resistance of the biobased resin.^36,62−64^

**Table 1:** Parameter Settings for the Synthesis of the Biobased Resin and VER Using Various Concentrations of the Cobalt Accelerator and Methyl Ethyl Ketone Peroxide (MEKP) Catalyst

entry	abbreviation	polyester	X500	X600	cobalt	MEKP	DC^a^	viscosity^b^
1^c^		2 g	2 mL					
2	ResCo_0.25_MEKP_2.25_	1 g	2 mL	2 mL	0.25 wt %	2.25 wt %	50%	0.31 Pa.s
3	ResCo_0.5_MEKP_2.25_	1 g	2 mL	2 mL	0.5 wt %	2.25 wt %	56%	5.8 Pa.s
4	ResCo_0.25_MEKP_4.5_	1 g	2 mL	2 mL	0.25 wt %	4.5 wt %	59%	1.3 Pa.s
5	ResCo_0.5_MEKP_4.5_	1 g	2 mL	2 mL	0.5 wt %	4.5 wt %	62%	4.7 Pa.s
6	ResCo_0.2_MEKP_4.5_	1 g	2 mL	2 mL	0.2 wt %	4.5 wt %	63%	0.42 Pa.s
7	ResCo_0.25_MEKP_5.0_	1 g	2 mL	2 mL	0.25 wt %	5.0 wt %	65%	0.43 Pa.s
8	VER-MEKP_5.0_				0.25 wt %	5.0 wt %	98%	9.6 Pa.s
9	VER-MEKP_4.5_				0.25 wt %	4.5 wt %	97%	7.8 Pa.s

a Degree of cross-linking (DC) was determined by comparing the FTIR spectra of the cured and uncured resin.

b The reported viscosity is obtained at a shear rate of 0.5 s^–1^.

c Using this concentration of polyester provided a highly viscous heterogeneous resin mixture. ResCo_0.25_MEKP_2.25_; Res = resin, Co_0.25_ = cobalt concentration, and MEKP_2.25_ = MEKP concentration.

**Figure 4:**
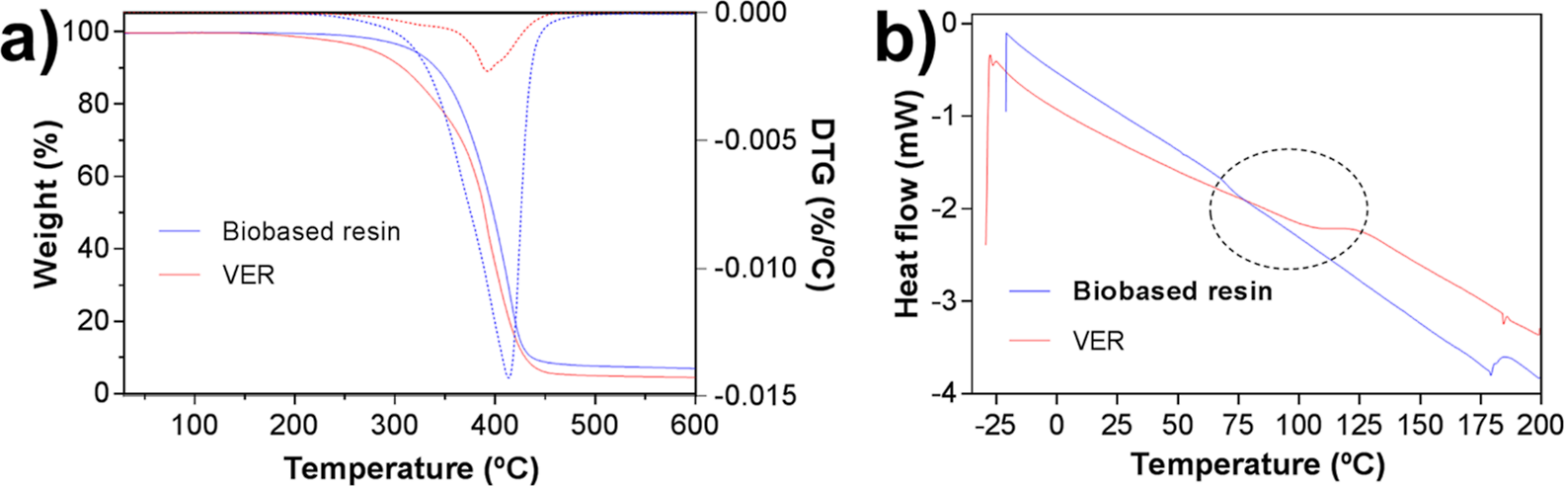
Thermal properties of the cured biobased resins and epoxy bisphenol A vinyl ester urethane (VER). (a) Representative selected TGA curves showing the weight and the dashed curves corresponding to the first derivative TGA. (b) DSC curves.

The thermal properties of the devised cured resins were evaluated through thermogravimetric analysis (TGA) and differential scanning calorimetry (DSC) analysis (Figure 4, Table 2). The starting degradation temperature (*T*_onset_) and temperature with the highest degradation rate (*T*_max_) were higher for the biobased resin (*T*_onset_ = 137.0–181.4 °C and *T*_max_ = 413.5–415.2 °C) (Table 2, entries 1–6) compared to the cured VER resin (*T*_onset_ = 129.8–132.1 °C and *T*_max_ = 393.2–392.8 °C) (Table 2, entries 7–8). The best biobased resin demonstrated the highest *T*_onset_ (181.4 °C) and *T*_max_ (415.2 °C) values (Table 2, entry 6). The residual at 600 °C was slightly higher for the cured biobased resin (7.1%) prepared according to the best of the studied parameter settings than VER (4.5 and 5.1%). However, the *T*_g_ was higher for the VER samples (VER: *T*_g_ = 101.9 and 104.2 °C and biobased resin: *T*_g_ = 74.1–99.6 °C). Despite that, the VER resin demonstrated a higher *T*_g_, and the biobased resin demonstrated better overall thermal stability and was more stable at higher temperatures. The results are also in accordance with the SI and GC analyses. A further increase in the DC of the biobased resin would most likely lead to a higher *T*_g_.^65^

**Table 2:** TGA and DSC Data for the Synthesized Resins

entry	sample	*T*_onset_ (°C)	*T*_max_ (°C)	*T*_final_ (°C)	residual at 600 °C	*T*_g_ (°C)
1	ResCo_0.25_MEKP_2.25_	150.2	413.5	450.1	9.3%	74.1
2	ResCo_0.5_MEKP_2.25_	150.5	414.7	455.2	5.7%	77.1
3	ResCo_0.25_MEKP_4.5_	137.0	414.0	447.3	6.4%	88.3
4	ResCo_0.5_MEKP_4.5_	155.1	414.0	445.0	5.3%	83.3
5	ResCo_0.2_MEKP_4.5_	144.5	414.0	457.7	6.7%	83.8
6^a^	ResCo_0.25_MEKP_5.0_	181.4	415.2	447.4	7.1%	99.6
7	VER-MEKP_4.5_	129.8	393.2	450.1	5.1%	101.9
8^b^	VER-MEKP_5.0_	132.1	392.8	453.5	4.5%	104.2

a The tensile strength, elongation at break, and Young’s moduli for this sample were 14.08 ± 7.11 MPa, 4.63 ± 1.09%, and 774.99 ± 199.85 MPa, respectively.

b The tensile strength, elongation at break, and Young’s moduli for this sample were 37.58 ± 4.72 MPa, 9.18 ± 1.53%, and 610.92 ± 67.66 MPa, respectively.

**Figure 5:**
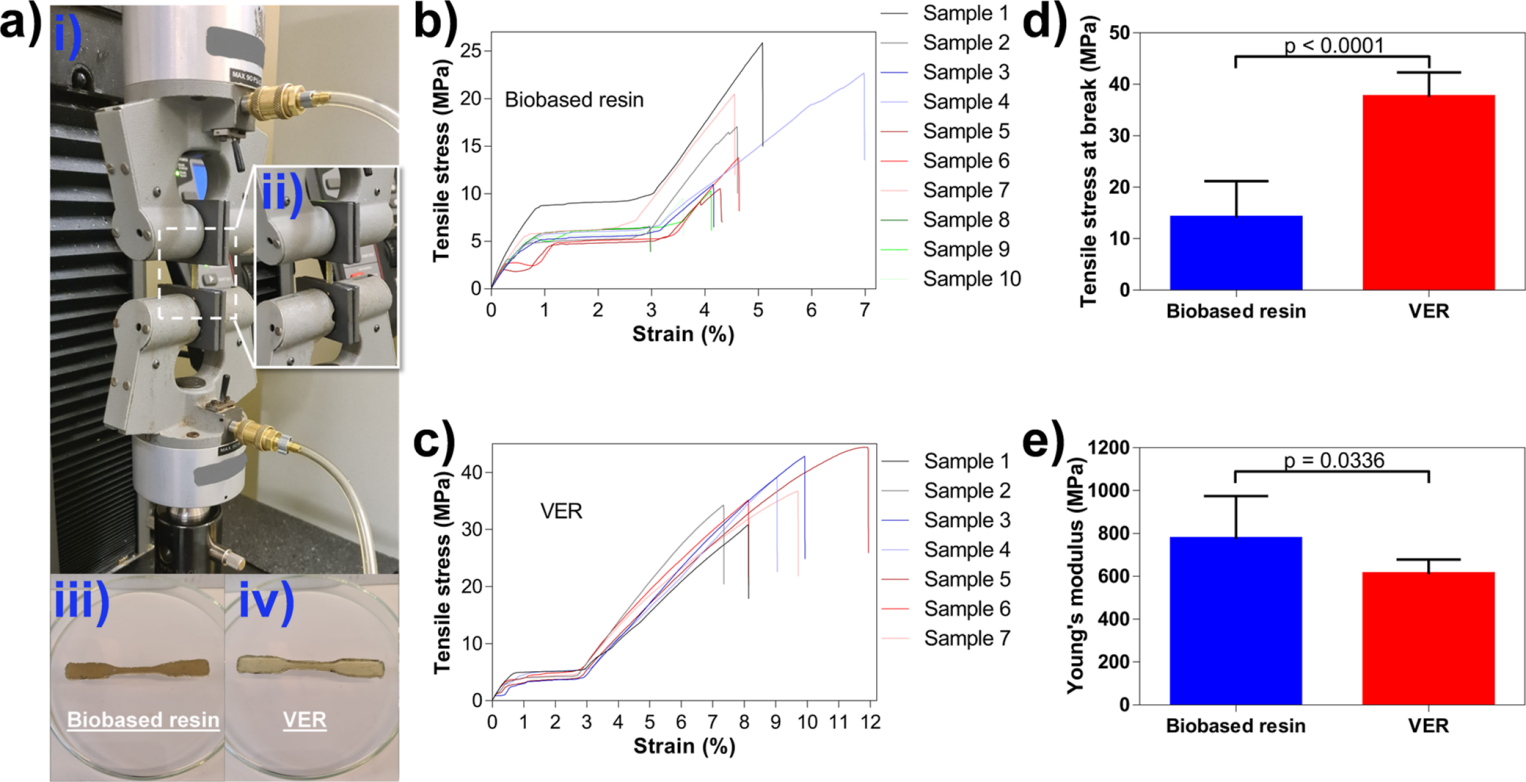
Tensile testing of the cured resins. (a) (i) Photo demonstrating the setup for the tensile test, (ii) gripper, (iii) photo showing a cured dog-bone-shaped biobased resin, (iv) photo showing a cured dog-bone-shaped VER resin, (b) stress–strain curves of cured biobased resin samples, (c) stress–strain curves of the cured epoxy bisphenol A vinyl ester urethane (VER) resin samples, (d) tensile stress at break of the cured resins, and (e) Young’s moduli of the cured resins. Values are means ± standard deviation (SD), *p*-values were calculated using the student’s *t*-test comparing the two groups, and *p* < 0.05 indicates significant differences.

The mechanical properties were evaluated by tensile testing (Figure 5a). The cured VER showed to withstand higher tensile stress, as demonstrated by the significant (*P* < 0.0001) higher tensile stress at break (37.58 ± 4.72 MPa) compared to the cured biobased resin (14.08 ± 7.11 MPa) (Figure 5b–d). However, the biobased resin was stiffer and thus more difficult to deform and needed higher force to deform, which is demonstrated in the significant (*P* < 0.0336) higher Young’s modulus obtained (biobased resin = 774.99 ± 199.85 MPa and VER = 610.92 ± 67.66 MPa) (Figure 5e). This trend could also be observed in the elongation at break data, where the cured biobased resin showed an elongation at break of 4.63 ± 1.09% and the cured VER resin was 9.18 ± 1.53%. The higher elongation at break for VER resin is an indication that the material is capable of stretching more, more ductile, more flexible, and tougher (Table 2).^66,67^ Moreover, similar trends were observed when the tensile testing was performed with a slower crosshead speed (2 mm/min), and no significant difference was observed in the Young’s modulus (Figure S5).

Overall, key performance properties put forth the synthesized FA/IA/2,5-FDCA/BD polyester as a feasible, curable, all-biobased resin competitive with the assessed commonly used fossil-based counterpart.

The findings in the presented work provide a promising and interesting future for continuing the design of an all-biobased tunable and curable resin for various industrial applications. For instance, future research endeavors within the presented topic would focus on further increasing the DC of the biobased resin to improve its properties, such as mechanical properties and *T*_g_. This could be accomplished, for instance, by further increasing the MEKP catalyst concentration during the curing, evaluating various temperatures for the curing, etc. Moreover, increasing the concentration of the monomer 2,5-FDCA within the polyester would most likely promote the overall mechanical performance of the biobased resin. Furthermore, increasing the concentration of the reactive viscosity enhancer relative to the reactive diluent might also impact the overall performance of the resin, which is therefore important to further investigate. Additionally, dynamic mechanical analysis (DMA), which is more sensitive, should be performed to get a more profound understanding of the mechanical performance.

## 3 Conclusions

An all-biobased solvent- and styrene-free curable resin is fabricated, comprising a polyester moiety and reactive diluents. The polyester is synthesized through a green acid-catalyzed solvent-free condensation reaction with rationally selected biobased monomers. The final curable resin is fabricated by combining the polyester with a reactive diluent and a reactive viscosity enhancer. These combinations would allow for further tuning of the viscosity of the final resin for a suitable application. The thermal and mechanical performance of the fabricated biobased resin was compared to the commercial epoxy bisphenol A vinyl ester urethane resin (VER) containing styrene (<50 wt %). The VER-based cured resin demonstrated higher *T*_g_ (up to 104.2 °C); nevertheless, the biobased cured resin demonstrated higher overall thermal stability with *T*_max_ up to 415.2 °C, starting degradation temperature <181.4 °C, and *T*_g_ up to 99.6 °C (VER: *T*_max_ up to 392.8 °C and *T*_onset_ up to 132.1 °C). The tensile stress of the cured VER resin was significantly higher (37.6 MPa) than that of the cured biobased resin (∼15.1 MPa); nevertheless, the biobased resin showed to be significantly stiffer with a Young’s modulus of ∼775 MPa (VER ∼ 611 MPa). Furthermore, the SI and GC for the cured biobased resin further corroborated its better thermal stability and chemical resistance. The entire process was conducted without the use of solvents, which is essential for improving the process’s climate footprint and advantageous for scaling up and industrial implementation. Instead, a biobased reactive diluent was shown to be a viable substitute. In conclusion, the presented rational design provided an all-biobased curable resin in the absence of any solvent, free from styrene, and with a tailorable viscosity suitable for industrial application.

## 4 Experimental Section

### 4.1 Materials

The following chemicals were purchased from Sigma-Aldrich: methacrylic anhydride (≥94.0%), 1,4-butanediol (BD, ReagentPlus, 99%), itaconic acid (IA, ≥99.0%), fumaric acid (FA, ≥99.0%), *p*-toluenesulfonic acid monohydrate (ACS reagent, ≥98.5%), 4-methoxyphenol (ReagentPlus, 99%), sodium hydroxide (NaOH, ACS reagent, ≥97.0%, pellets), potassium permanganate (KMnO_4_, ACS reagent, ≥99.0%), cobalt(II) 2-ethylhexanoate solution (65 wt % in mineral spirits), and 2-butanone peroxide [methyl ethyl ketone peroxide (MEKP)]. 5-(Hydroxymethyl)furan 2-carbaldehyde (98%) was purchased from AmBeed (Arlington, IL, USA). X500 (diacrylate-based material) and X600 (dimethacrylate-based material) were used as received from the supplier (Sigma-Aldrich). VER was gracefully received as a gift from AOC Nederland B.V.

### 4.2 Synthesis of 2,5-Furandicarboxylic Acid (2,5-FDCA)

The 2,5-FDCA was prepared following the method in a previous report with minor modifications.^68^ A round-bottomed flask was charged with NaOH (73 g, 1.84 mol) and dissolved by the addition of H_2_O (30 mL). Subsequently, 5-(hydroxymethyl) furan 2-carbaldehyde (10.1 g, 0.08 mol) and then KMnO_4_ (29.1 g, 0.184 mol) were added. The mixture was stirred for 30 min at room temperature, and then, the solid KMnO_4_ was filtered off. Afterward, HCl (concentrated) was added to the solution dropwise to keep the pH at 1.0 or less. The precipitated product was filtered off, washed with deionized water, and dried at 60 °C for 24 h, providing the 2,5-FDCA product (6.4 g, 51% yield) as a light brown powder.

### 4.3 Synthesis of Unsaturated Polyester

The unsaturated polyester was synthesized following the method in a previous report with minor modifications.^41^ The biobased polyester was synthesized by mixing the monomers IA (8.46 g, 65 mmol, 1.0 equiv), FA (6.79 g, 58.5 mmol, 0.9 equiv), 2,5-FDCA (1.02 g, 6.5 mmol, 0.1 equiv), and BD (13.8 mL, 156 mmol, 2.4 equiv); the catalyst *p*-toluenesulfonic acid (100 mg, 0.53 mmol, 0.008 equiv); and the radical inhibitor 4-methoxyphenol (100 mg, 0.81 mmol, 0.012 equiv) in a round-bottomed flask. Subsequently, the mixture is heated to 160 °C under stirring and purging with N_2_-gas during the entire reaction time. After 7 h, the reaction is cooled to room temperature and subsequently diluted with dichloromethane (as small an amount as possible). Next, the solution is poured into a beaker containing cold methanol, and the product immediately precipitates. The precipitate is filtered off, washed with methanol, and dried under vacuum, providing the product (13.3 g) as an off-white powder.

### 4.4 Representative Example for the Preparation and Curing of the Biobased Resin

The biobased resin was prepared by mixing the polyester (2.5 g) in the reactive diluent X500 (5.6 g, 5.0 mL) and the reactive viscosity enhancer X600 (5.3 g, 5.0 mL) under sonication until fully dissolved and homogeneous. Subsequently, cobalt(II)-ethylhexanoate (33 mg, 33 μL) was added and mixed. Next, the MEKP (660.8 mg, 630 μL) was added to the mixture and immediately mixed until homogeneous. Afterward, the mixture was kept under a nitrogen (N_2_) atmosphere for 5 h for curing to complete.

### 4.5 Fourier Transform Infrared Spectrometry

FTIR analysis was carried out on a PerkinElmer Spectrum 100 FT-IR Spectrometer equipped with a single reflection [attenuated total reflection (ATR)] accessory unit (Golden Gate) from Graseby Specac LTD (Kent, England) and a TGS detector using the Golden Gate setup. The spectra were collected based on 16 scans averaged in the transmittance mode at regions between 4000 and 600 cm^–1^ and with 4 cm^–1^ resolutions. Data were recorded and processed using the software PerkinElmer Spectrum (2015). The degree of conversion (DC) corresponds to the percentage of C=C bonds converted to single bonds (C–C) during curing to form the polymer resin. The curing of the biobased resin was calculated by following the decrease of the peak centered at 1637 or 813 cm^–1^ corresponding to the C=C stretching vibration. To quantify the DC, the peak at 1714 cm^–1^ representing the C=O stretching vibration was taken as internal standard since it is not affected by the curing process. For the VER resin, the peak at 1720 cm^–1^ corresponding to the C=O or the aromatic C=C bond at 1601 cm^–1^ was taken as internal standards since these are not affected by the curing process and following the decrease of the peak centered at 1630 cm^–1^. The calculations were performed following eq 1(69)
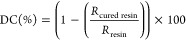
1

### 4.6 Differential Scanning Calorimetry

DSC analysis was performed using a Mettler-Toledo DSC1 STARe system. Samples having masses of ≈6 mg were inserted in 100 μL aluminum pans with pierced lids. The applied heating rate was 10 °C/min in a N_2_-atmosphere (rate 50 mL/min). The thermal behavior of the samples was investigated by using two repeated heating–cooling cycles. The temperature program was as follows: the temperature was first ramped from 25 to 200 °C and kept at 200 °C for 2 min, followed by a cooling cycle from 200 to −30 °C. After an isotherm at −30 °C for 2 min, a second heating cycle was performed from −30 to 200 °C. The glass transition temperature (*T*_g_) was determined from the second heating curve. Data analysis was performed on Mettler STARe evaluation software.

### 4.7 Thermogravimetric Analysis

TGA was performed with a Mettler-Toledo TGA/SDTA 851e instrument. Samples having masses of ≈8 mg were used, and the experiment was performed at a heating rate of 10 °C/min under an N_2_-atmosphere with a purge rate of 50 mL/min at the temperature range 30–600 °C. The samples were kept isothermally at 120 °C for 10 min to remove solvent residues and then cooled to 30 °C, followed by heating at 600 °C, and the starting degradation temperature (*T*_onset_), temperature with the highest degradation rate (*T*_max_), final degradation temperature (*T*_final_), and residual amount at 600 °C were determined. Data analysis was performed on Mettler STARe evaluation software.

### 4.8 Proton Nuclear Magnetic Resonance Spectroscopy (^1^H NMR)

^1^H NMR analysis was performed at 298 K on a 400 MHz Bruker Avance III HD spectrometer using deuterated chloroform (CDCl_3_) or deuterium oxide (D_2_O) as the solvent. Spectra were based on 128 scans and reported in parts per million relative to the solvent residual peak at 7.26 ppm for CDCl_3_ or at 4.79 ppm for D_2_O. MestReNova 9.0 software was used for data analysis.

### 4.9 Size Exclusion Chromatography

SEC analysis to determine the molecular weight was performed on a Malvern GPCMAX instrument equipped with an autosampler, a PLgel 5 μm guard column (7.5 mm × 50 mm), and two PLgel 5 μm MIXED-D (300 mm × 7.5 mm) columns. The polymer sample (4–5 mg/mL) was dissolved in chloroform containing 2% v/v toluene, which was also used as an eluent. The flow rate was 0.5 mL/min, and the temperature was kept at 35 °C. Narrow disperse polystyrene standards with molecular weights in the range of 1200–400,000 g/mol were used for calibration. OmniSEC version 5.10 software was used for data analysis.

### 4.10 Tensile Testing

Mechanical testing was performed on an Instron 5944 Universal Testing Machine with a single column, a 2 kN load cell, and a crosshead speed of 10 and 2 mm/min. The tensile test was performed according to the standard ASTM D638 using dog-bone-shaped specimens with nominal dimensions of 60 × 10 × 1 mm. All of the samples were conditioned at 23 °C and 50% relative humidity for 2 days before testing. The results of the tensile tests were based on the average of 10 (for the biobased resin) and 7 (for VER) individual measurements. The tensile stress-at-break was obtained at the failure point, and Young’s modulus was determined from the slope in the linear region of the stress–strain curve. Bluehill software was used for the test control and data acquisition.

### 4.11 SI and GC

The SI and GC were measured following the methods in a previous report with minor modifications.^62^ Three separate samples from each group (∼150 mg) were immersed in THF for 24 h separately. Subsequently, the mass of each sample was determined after swelling in THF. The SI was calculated following eq 2, where *m*_1_ is the mass of the material after swelling, and *m*_2_ is the initial mass of the samples
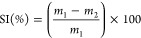
2

Next, the samples were dried in an oven at 70 °C for 24 h. The GC was calculated following eq 3, where *m*_3_ is the mass of the samples after oven drying, and *m*_2_ is the initial mass of the samples.
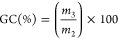
3

### 4.12 Viscosity Measurements

The viscosity was determined by rheological analysis with a TA Instrument model DHR-2. About 0.300 mL of the samples were used for the tests. The viscosity was recorded using a parallel plate of plate steel (25 mm diameter) with a gap of 100 μm and by performing a shear rate sweep at 0.1–1000 s^–1^ with 10 points/decade). The viscosity was determined at an initial shear rate of 0.5 s^1^.

### 4.13 Statistical Analysis

The experiments were analyzed using the Student’s *t*-test and compared the means of the two groups. The software GraphPad Prism 6 was used to calculate the statistics. Values are means ± standard deviation (SD), where *p* < 0.05 indicates significant differences and ns = no significance.

## Supporting Information Available

^1^H NMR spectra of itaconic acid, fumaric acid, and 2,5-furandicarboxylic acid and stress–strain curves, tensile stress at break, Young’s moduli, and elongation at break of the cured VER and biobased resin (PDF)
