# Supplementary material for: Engineering an All-Biobased Solvent- and Styrene-Free Curable Resin
Source: ACS Polym Au. 2023 Oct 3;3(6):447–56. doi: 10.1021/acspolymersau.3c00015 (PMC10722568; doi:10.1021/acspolymersau.3c00015)
Supplement: Supplementary file 1 — lg3c00015_si_001.pdf [file lg3c00015_si_001.pdf]

Supporting Information

Engineering an All-Biobased Solvent- and Styrene-Free Curable Resin

*Samson Afewerki * and Ulrica Edlund **

Fibre and Polymer Technology, KTH Royal Institute of Technology, SE 100 44 Stockholm, Sweden

Supplementary Figures

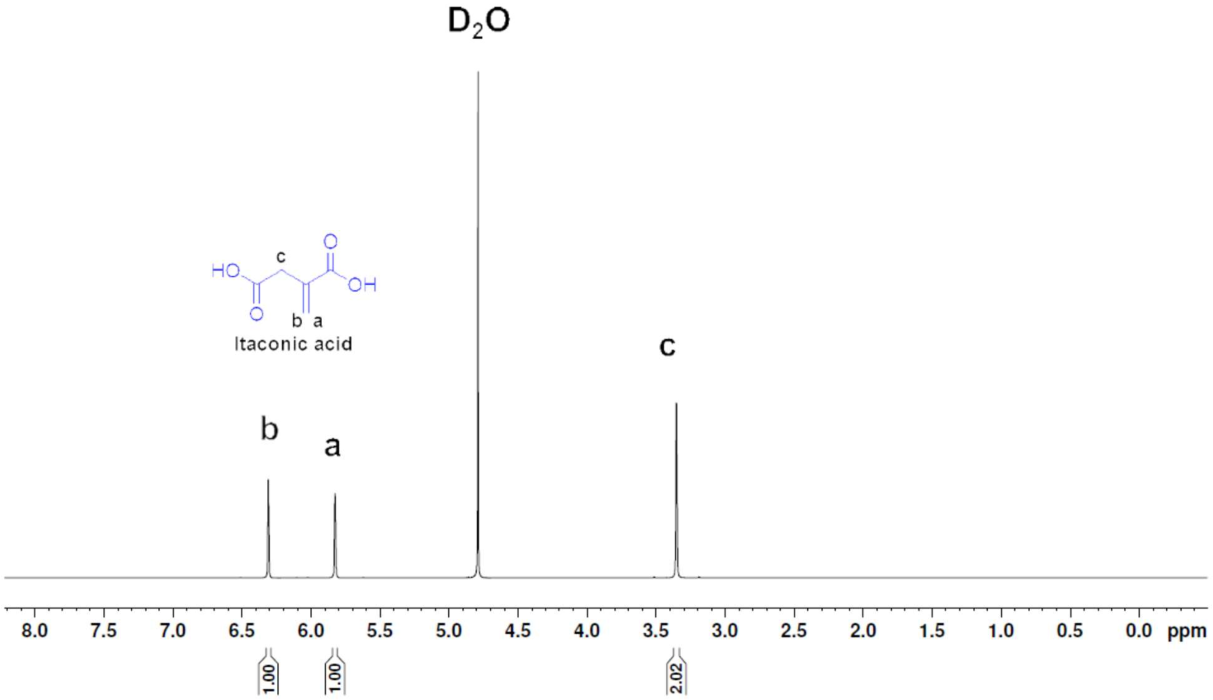

Figure S1. ^{1}H-NMR spectrum of Itaconic acid monomer.

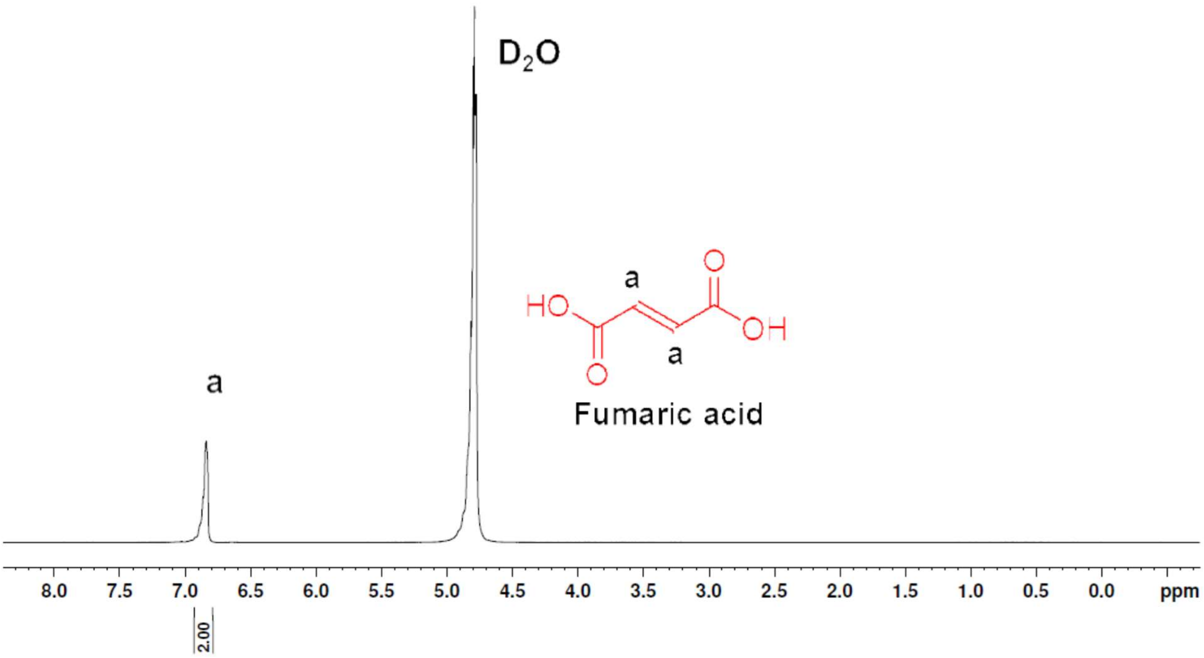

Figure S2. ^{1}H-NMR spectrum of Fumaric acid monomer.

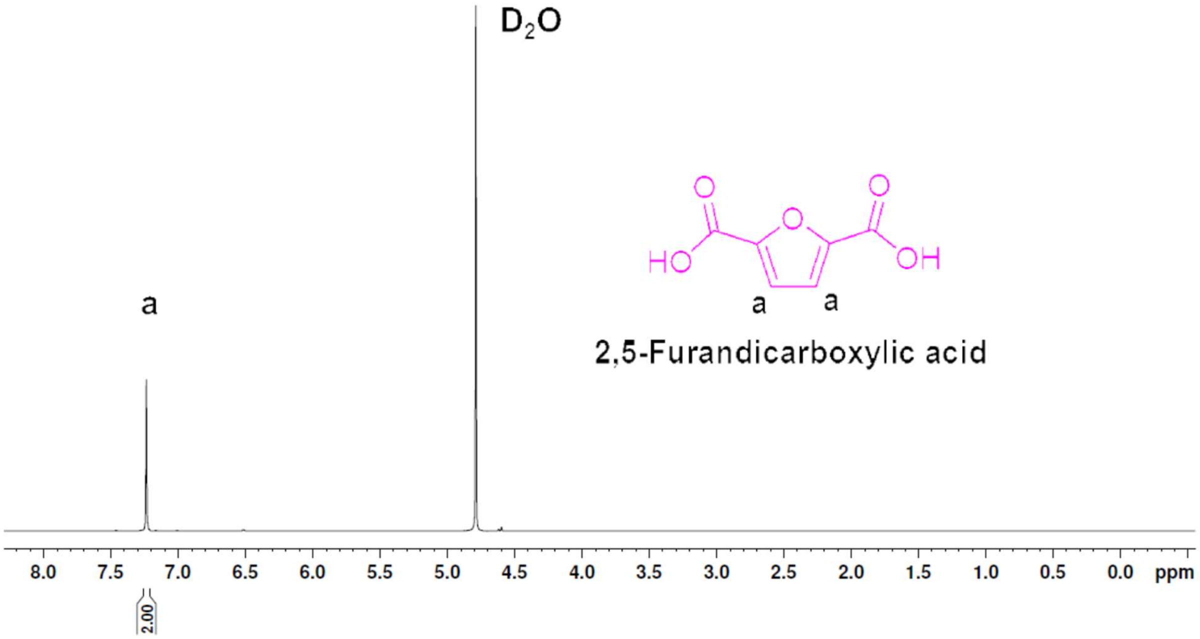

Figure S3. ^{1}H-NMR spectrum of 2,5-Furandicarboxylic acid monomer.

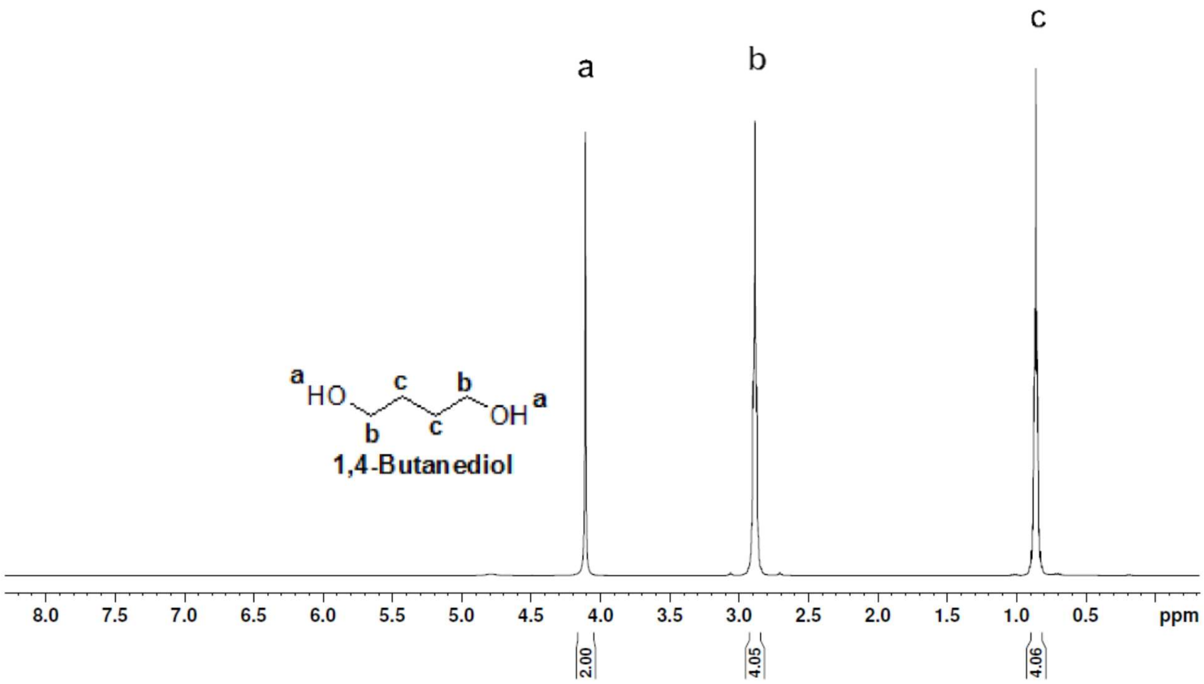

Figure S4. ^{1}H-NMR spectrum of 1,4-Butanediol monomer.

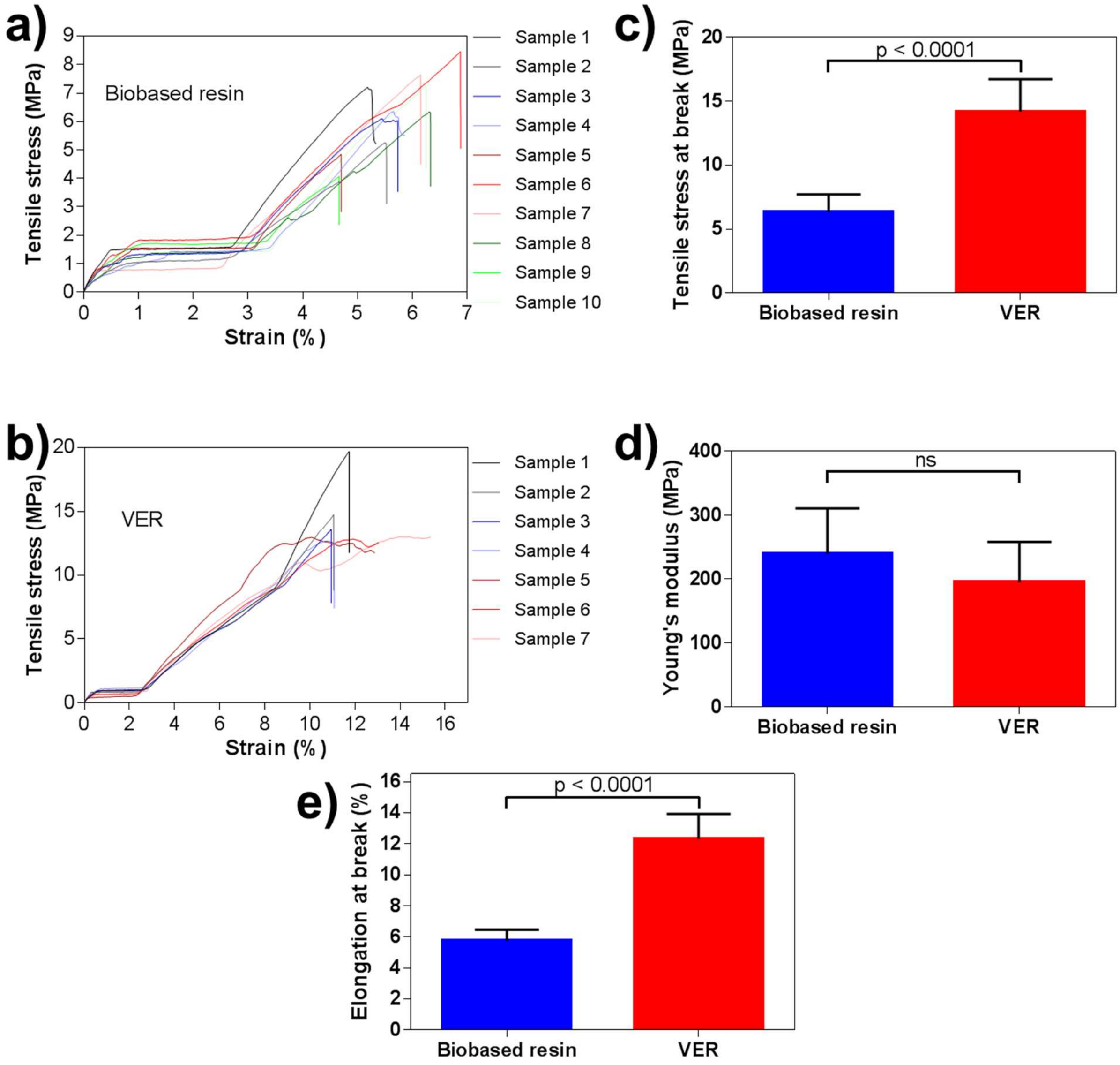

Figure S5. Tensile testing of the cured resins with a crosshead speed of 2 mm/min. **a)** Stress-strain curves of the biobased resin. **b)** Stress-strain curves of the VER resin. **c)** Tensile stress at break. **d)** Young's moduli. **e)** Elongation at break. Values are means ± standard deviation (SD), p-values were calculated using the student's t-test comparing the two groups, $p<0.05$ indicating significant differences, and ns = no significance.
